# The utility of routine surveillance screening with magnetic resonance imaging (MRI) to detect tumour recurrence in children with low-grade central nervous system (CNS) tumours: a systematic review

**DOI:** 10.1007/s11060-018-2901-x

**Published:** 2018-06-09

**Authors:** Simon P. Stevens, Caroline Main, Simon Bailey, Barry Pizer, Martin English, Robert Phillips, Andrew Peet, Shivaram Avula, Sophie Wilne, Keith Wheatley, Pamela R. Kearns, Jayne S. Wilson

**Affiliations:** 1 Cancer Research UK Clinical Trials Unit (CRCTU), Institute of Cancer and Genomic Sciences, University of Birmingham, Birmingham, UK; 2 Sir James Spence Institute of Child Health, Royal Victoria Infirmary, Newcastle upon Tyne, UK; 3 Alder Hey Children’s NHS Foundation Trust, Liverpool, UK; 4 Institute of Cancer and Genomic Sciences, University of Birmingham, Birmingham, UK; 5 Birmingham Women and Children’s Hospital NHS Foundation Trust, Birmingham, UK; 6 Centre for Reviews and Dissemination (CRD), University of York, York, UK; 7 Queen’s Medical Centre, Nottingham University Hospitals’ NHS Trust, Nottingham, UK

**Keywords:** Systematic review, Magnetic resonance imaging (MRI), Surveillance, Children, Central nervous system (CNS) tumours, Recurrence, Pilocytic astrocytoma, Low grade glioma

## Abstract

**Background:**

Magnetic resonance imaging (MRI) is routinely used as a surveillance tool to detect early asymptomatic tumour recurrence with a view to improving patient outcomes. This systematic review aimed to assess its utility in children with low-grade CNS tumours.

**Methods:**

Using standard systematic review methods, twelve databases were searched up to January 2017.

**Results:**

Seven retrospective case series studies (n = 370 patients) were included, with average follow-up ranging from 5.6 to 7 years. No randomised controlled trials (RCTs) were identified. Due to study heterogeneity only a descriptive synthesis could be undertaken. Imaging was most frequent in the first year post-surgery (with 2–4 scans) reducing to around half this frequency in year two and annually thereafter for the duration of follow-up. Diagnostic yield ranged from 0.25 to 2%. Recurrence rates ranged from 5 to 41%, with most recurrences asymptomatic (range 65–100%). Collectively, 56% of recurrences had occurred within the first year post-treatment (46% in the first 6-months), 68% by year two and 90% by year five. Following recurrence, 90% of patients underwent treatment changes, mainly repeat surgery (72%). Five-year OS ranged from 96 to 100%, while five-year recurrence-free survival ranged from 67 to 100%. None of the studies reported quality of life measures.

**Conclusion:**

This systematic review highlights the paucity of evidence currently available to assess the utility of MRI surveillance despite it being routine clinical practice and costly to patients, their families and healthcare systems. This needs to be evaluated within the context of an RCT.

**Electronic supplementary material:**

The online version of this article (10.1007/s11060-018-2901-x) contains supplementary material, which is available to authorized users.

## Introduction

Paediatric low-grade CNS tumours are an extremely diverse group of neoplasms. The likelihood of recurrence is largely a function of tumour type, but also varies according to tumour location as well as treatment regimens [1].

Surveillance neuroimaging is routinely used to detect recurrence in children with low-grade CNS tumours in the absence of clinical signs and symptoms. The rationale behind surveillance is that recurrence detected at a stage when there is less disease will be more responsive to treatment and this will result in improved outcomes for patients. The scheduling and imaging techniques employed, or surveillance protocols, are loosely based on the biological characteristics of the different CNS tumour types, taking into account the rate of tumour growth, location and patterns of local and metastatic recurrence [2, 3].

In recent years, magnetic resonance imaging (MRI) has replaced computed tomography (CT) as the dominant surveillance neuroimaging modality. Its greater imaging resolution and multi-planar capability account for its superior diagnostic utility, particularly with respect to soft tissue neoplasms such as CNS tumours [4]. However, despite being standard practice, there have been no systematic reviews assessing surveillance MRI in this patient group.

The aims of this systematic review were to:

1. Assess the utility of surveillance MRI to detect early tumour recurrence in children with no new, stable or improved neurological signs or symptoms with low-grade CNS tumours compared to the use of non-routine imaging undertaken on presentation with disease signs or symptoms and whether this results in improved clinical outcomes for patients and their families;
2. Evaluate the effects of varying MRI screening intervals across tumour types and determine the optimum length of time for screening post-initial diagnosis;
3. Identify gaps and methodological weaknesses in the current evidence base to inform the design of future studies.

## Methods

This review is part of a series of systematic reviews looking at treatments for paediatric CNS tumours. The project included public and patient involvement (PPI), consisting of the parents of children with CNS tumours who expressed a particular interest in this review question, which was pivotal in our decision to undertake the current review.

Standard systematic review methodology aimed at minimising bias was employed and reporting followed the Preferred Reporting Items for Systematic Reviews and Meta-Analyses (PRISMA) guidelines [5]. A detailed account of the methodology employed in this review can be found in the published protocol, which is also registered with PROSPERO (CRD42016036802) [6].

### Search strategy

Searches for published studies from 1985 to January 2017 were undertaken in a number of databases, including MEDLINE, EMBASE and the Cochrane Library. No language/publication restrictions or study design filters were applied (see Online Resource 1 for search strategy and databases searched). Reference lists of included studies were citation-checked and experts in the field consulted for published studies not retrieved by the electronic searches.

### Study selection

The following inclusion and exclusion criteria were applied:

*Population* Children and young adults (age up to 25 years) with diagnoses of any type of low grade (i.e. WHO grade I and II) CNS tumour who had either no new, stable or improved neurological signs or symptoms at the time of study recruitment.

*Intervention* Routine interval follow-up MRI scans conducted at any screening interval determined within the primary study. Studies reporting CT scans were excluded.

*Outcomes* Outcome measures included recurrence rates (by study, tumour type, location and extent of resection), diagnostic yield of imaging, timing of recurrence, change in patient management post-recurrence, overall survival (OS), surrogate survival measures (i.e. recurrence-free survival (RFS) and progression-free survival (PFS)) and quality of life.

Patients were deemed to have experienced a recurrence if, after undergoing complete surgical removal of their primary tumour [i.e. achieving a gross total resection (GTR)], evidence of tumour was captured on a subsequent MRI scan. Patients were deemed to have experienced progressive disease if, after undergoing incomplete surgical removal of their primary tumour [i.e. achieving a sub-total resection (STR)], evidence of a significant increase in the size of the tumour was captured on a subsequent MRI scan. However, for the purposes of this paper, we use the term ‘recurrence’ to cover both recurrence in GTR and progression in STR patients.

All of the studies in this review reported patient outcomes in terms of whether or not patients were asymptomatic at recurrence. However, children undergoing surveillance imaging for detection of recurrent disease will often be asymptomatic from the recurrence but may have some neurologic sequelae from their tumour and/or its treatment. This was the reason for characterising patients as having either no new, stable or improved neurological signs or symptoms.

*Study designs* Randomised controlled trials (RCTs) and non-randomised comparative studies were initially sought. However, as no such studies were identified the review was extended to include observational studies, such as case series studies. Single case reports, letters or editorials were excluded.

Study selection was undertaken by two independent reviewers. Citations marked for inclusion on the basis of title and abstract underwent full text assessment. Disagreements were resolved by discussion (see Online Resource 2 for details of excluded studies).

### Data extraction and risk of bias assessment

Data was extracted by one reviewer and checked by a second and recorded on a standardised pro-forma (see Online Resource 3). Risk of bias was conducted by two reviewers and assessed at the study level using a six-point tool devised by the Centre for Reviews and Dissemination (York; CRD) specifically designed to assess bias in case series studies [7].

### Statistical analysis

Due to the design of the studies included in the review and the heterogeneity of outcomes reported, only a descriptive analysis of the data was possible.

## Results

### Quantity of the research

**Fig. 1:**
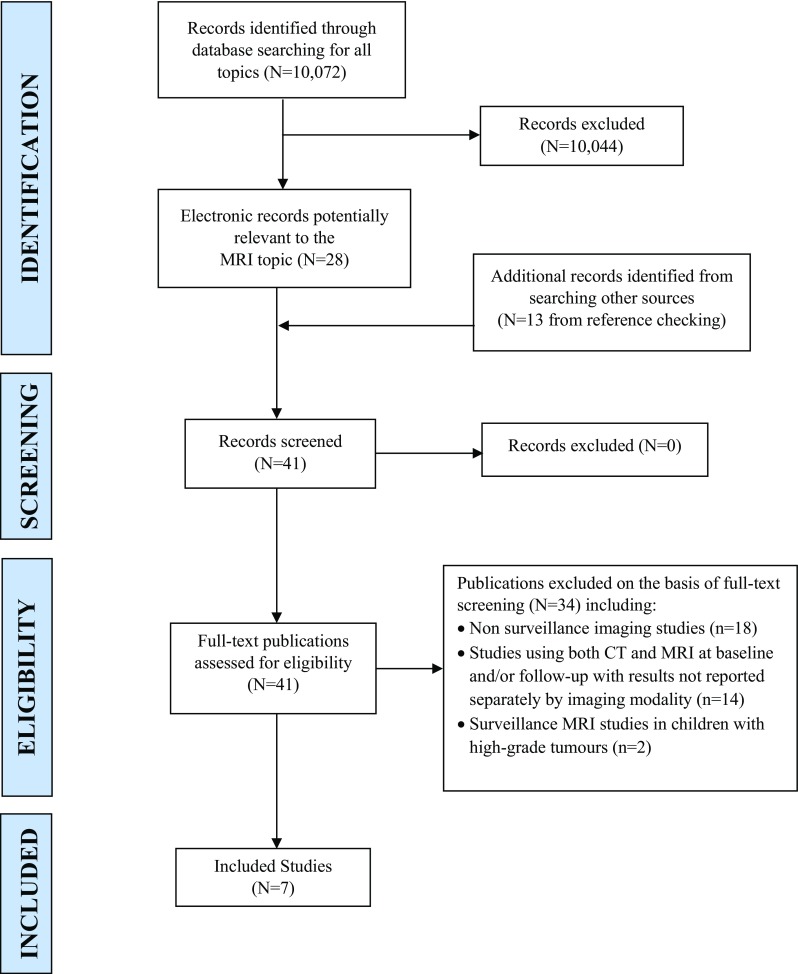
PRISMA diagram of flow of studies through the selection process

From the electronic database searches, 28 publications were considered potentially relevant to this review, with an additional 13 identified from citation checking. On full text examination, 34 publications did not meet the inclusion criteria including 14 surveillance imaging studies which employed both CT and MRI but failed to report results separately for MRI. No RCTs or prospective comparative studies were identified. Seven retrospective case series were included in the review [8–14] (see Fig. 1 for the PRISMA flow diagram).

### Quality of the research

All seven studies were undertaken at single centre institutions. Study samples were small but appeared to be representative of the relevant population, i.e. children with low-grade CNS tumours undergoing surveillance imaging using MRI to detect recurrence. In all studies, inclusion and exclusion criteria for each study were explicitly stated. Generally, participants were at a similar time point in the course of their disease in that all had undergone resection of their primary tumour, whether a GTR or STR. Definitions of GTR and STR were provided although definitions varied between studies (see Online Resource 4 for definitions table). No studies containing STR patients reported the size/extent of residual tumour. Duration of follow-up was reported in five studies [9, 11–14]. Assessment of outcomes using objective criteria was variable. The definition for radiographic recurrence was provided in all but two studies [10, 12] but again definitions varied between studies. All but one study [13] reported details of the change in patient management/treatment post-recurrence. Definitions of survival outcome measures were provided in only half of the studies reporting survival, with one study failing to define OS [8] and one RFS [12] (see Online Resource 5 for quality assessment table).

### Description of included studies

The seven studies were published between 2001 and 2016 with six published since 2009 [9–14]. Five studies were undertaken in the USA [8, 9, 11–13], one in Canada [14] and one in Australia [10]. The studies (overall n = 469 patients) were comprised of six low grade tumour studies [9–14] and one with a mix of low and high-grade tumours [8]. The total number of patients in the low-grade studies was 357 with a mean sample size of 60. Mean age at diagnosis was 7.5 years, 48% of patients were male and 75% of tumours were located in the posterior fossa. Median follow-up ranged from 5.6 to 7 years. Three studies [9, 11, 13] explicitly excluded children with Neurofibromatosis Type 1, with the remaining studies providing no details of the NF1 status of their study populations. At the commencement of surveillance imaging, none of the patients had relapsed disease (See Table 1).

**Table 1:** Study characteristics of included low-grade tumour studies

Study (year) [ref] Location Years of study Study design	Aim	Population	Intervention	Outcomes reported
Alford et al. [9] USA 2000–2013 Retrospective case series	To test hypothesis that PA pts without residual tumour after surgery and with > 2 consecutive, negative surveillance MRI scans, are unlikely to suffer a recurrence thereafter and that therefore further surveillance imaging is unnecessary	**Included**: Pts with cerebellar PA (WHO grade 1) without brainstem involvement and at least 2 years postoperative MRI follow-up **Excluded**: pilomyxoid astrocytoma (WHO grade 2), pts with NF1 and those without preoperative imaging **N** = 53 (43% male) **Tumour location**: cerebellum (posterior fossa) **Median age at diagnosis**: 6.69 years (range 1.50–18.99) **Median follow-up**: 6.05 years (2.07–12.28) **Previous treatments:** GTR/indeterminate: n = 41 (77%) STR: n = 12 (23%)	Surveillance MRI: 1.5T (pre-2005) and 1.5T/3T (post-2005) magnets; Diffusion-weighted imaging using spin-echo sequence Image sequences: T1-weighted, FLAIR and T2-weighted; Gd-enhanced T1 weighted and FLAIR **Average frequency of imaging**: every 3 months for first 2 years, every 6 months in 3rd year and every 12 months thereafter **Median number of images per pt**: n = 9 (GTR/indeterminate pts only)	Median no. of surveillance images per pt (GTR pts only) Median time to recurrence/progression Recurrence rate Diagnostic yield of MRI
Dodgshun et al. (2016) [10] Australia 1996–2013 Retrospective case series	To determine the optimal management in paediatric pilocytic astrocytoma post-surgery, with a view to proposing a restricted schedule of MRI surveillance resulting in time and cost savings	**Included**: pts with PA (grade unspecified) who underwent GTR **Excluded**: pts who underwent adjunctive therapy post-resection **N** = 67 (55% male) **Median age at diagnosis**: 6.9 years (range 1–16) **Average follow-up**: NR although 33 patients (49%) had at least 5 years from diagnosis **Tumour location:** Posterior fossa: n = 58 (87%) Supratentorial: n = 9 (13%) **Previous treatments:** GTR: n = 67 (100%)	Surveillance MRI: details of MRI scanner and image sequences NR **Average frequency of Imaging: NR** **Average number of MRI images per pt** NR for whole cohort for study period 12 (mean; range 7–20 scans) based on 33 pts with minimum 5 years follow-up	Mean time to recurrence/progression from diagnosis Recurrence rate Changes in pt treatment post-recurrence OS EFS
Dorward et al. (2010) [11] USA NR Retrospective case series	To create a post-operative surveillance imaging strategy that both emphasizes the initial postoperative MRI as a baseline and incorporates histopathological variables for determining the optimal surveillance imaging interval for posterior fossa pilocytic astrocytoma	**Included**: pts with PA (WHO grade 1) located in the posterior fossa with clinical and > 2 years radiographic follow-up **Excluded**: pts with NF1 **N** = 40 (45% male) **Mean age at diagnosis**: 8 years (range 1.4–19.9) **Mean follow-up**: 5.6 years (2.1–19.8) **Previous treatments**: GTR: n = 40 (100%)	Surveillance MRI: 1.5T magnet Image sequences T1- and T2-weighted (± Gd contrast) **Average frequency of imaging**: every 3–6 months post-surgery, then at 1-year intervals post-surgery for duration of follow-up **Average number of images per pt**: NR	Median time to recurrence RFS by evidence of nodular enhancement on initial surveillance MRI
Kim et al. (2014) [12] USA 1993–2003 Retrospective case series	To employ MRI imaging to evaluate how often tumours recur and to determine if recurrences are associated with any clinical symptoms in children with conclusive evidence GTR; to propose guidelines regarding the frequency of post-surgery surveillance MRI imaging; to estimate the financial costs of imaging	**Included**: Pts with WHO Grade 1 glial and glioneuronal tumours with GTR and follow-up clinical and MRI data **Excluded**: NR **N **= 67 (42% male) **Mean age at surgery**: 9.1 years (range 1–21.5) **Mean follow-up**: 6.6 years (1-14.7) **Tumour type**: PA: n = 46 (69%) Ganglioglioma: n = 14 (21%) DNET: n = 6 (9%) Glioneuronal: 1% **Tumour location**: Cerebellum: n = 41 (61%) Temporal: n = 16 (24%) Parietal: n = 4 (6%) Frontal: n = 2 (3%) Brainstem: n = 2 (3%) Occipital: n = 2 (3%) **Previous treatments**: GTR: n = 67 (100%)	Surveillance MRI: details of magnet and image sequences NR **Average frequency of imaging**: Immediately in the postoperative period, every 3 months in year 1, every 6 months in year 2, yearly until 5 years post-surgery, and then every 2–3 years thereafter **Average number of MRI images per pt** 8.6 scans (mean) 9 scans (median; range 2–20 scans)	Median time to recurrence Recurrence rate by tumour type and location RFS post-resection (2- and 5-year) for whole cohort and by tumour type Changes in pt treatment post-recurrence
Udaka et al. (2013) [13] USA 1994–2010 Retrospective case series	To determine the clinical and radiographic characteristics associated with recurrent/progressive disease in children with LGG and to address the role and optimal frequency of surveillance MRI imaging in asymptomatic cases of paediatric LGG based on an evaluation of the timing of recurrence/progression	**Included**: pts with pathologically proven LGG (WHO Grade 1 or 2) **Excluded**: Pts with NF1/NF2, tuberous sclerosis complex, optic pathway glioma or brainstem glioma for which no pathological diagnosis was obtained **N** = 102 (52% male) **Median age at diagnosis**: 7 years (range 0.08 to 17) **Average follow-up**: NR **Tumour type**: PA: n = 76 (75%) Ganglioglioma: n = 8 (8%) Optic glioma: n = 7 (7%) DFA: n = 5 (5%) Oligodendroglioma: n = 3 (3%) Other astrocytoma: n = 3 (3%) Tumour location Posterior fossa: n = 49 (48%) Cortical: n = 15 (15%) Multifocal: n = 14 (14%) Basal ganglia/thalamus/hypothalamus n = 7 (7%) Optic tract/chiasmatic: n = 6 (6%) Tectal: n = 4 (4%) Other: n = 7 (7%) **Previous treatments**: Surgery only: n = 61 (59%) Surgery + ChemT: n = 20 (20%) Surgery + RT: n = 10 (10%) Surgery + ChemT + RT: n = 11 (11%) **Details of Surgery**: GTR: n = 38 (37%) STR/biopsy: n = 64 (63%)	Surveillance MRI: 1.5T magnet Image sequences: T1-weighted pre- and post-Gd, T2-weighted pre-Gd and diffusion-weighted **Average frequency of imaging** (n = 46 recurrent pts) 1 scan every 3.5 months (asymptomatic pts only) 1 scan every 3.6 months (asymptomatic pts only) **Average number of images per pt** 3.4 per year (irrespective of symptomatic status at recurrence)	Mean time to recurrence/progression OS PFS
Vassilyadi et al. (2009) [14] Canada 1987–2007 Retrospective case series	To evaluate the utility of ‘MRI surveillance strategy to detect recurrence or progression in children with pilocytic and non-pilocytic cerebellar astrocytoma	**Included**: pts with a histopathological diagnosis of a posterior fossa brain tumour (i.e. PA or non-PA cerebellar astrocytoma) **Excluded**: pts with incomplete chart information precluding collection of any follow-up information **N** = 28 (47% male) **Median age at surgery**: PA (n = 15): 7 ± 4 years (range 2–13) Non-PA (n = 13): 7 ± 5 years (range 1–15) GTR (n = 19): 7 ± 4 years STR (n = 9): 8 ± 4 years **Average follow-up**: PA (n = 15): 6 ± 3 years Non-PA (n = 13): 7 ± 5 years GTR (n = 19): 7 years STR (n = 9): 4.4 years **Tumour type**: PA: n = 15 (54%) Non-PA (diffuse fibrillary astrocytoma): n = 13 (46%) **Previous treatments**: GTR: n = 19 (68%) *PA: n* = *11 (58%)* *Non-PA: n* = *8 (42%)* STR: n = 9 (32%) *PA: n* = *4 (44%)* *Non-PA: n* = *5 (56%)*	Surveillance MRI: Details of magnet NR Image sequences: T1, T1 with Gd **Average frequency of imaging**: GTR pts (n = 19): 1 every 11 months STR pts (n = 9): 1 every 6 months **Average number of images per pt** PA: 7 ± 2 *GTR: 7* ± *2 (42% with general anaesthetic)* *STR: 7* ± *2 (55% with general anaesthetic)* Non-PA: 8 ± 4 *GTR: 8* ± *5 (47% with general anaesthetic)* *STR: 9* ± *4 (47% with general anaesthetic)*	Average number of images per pt by tumour type and extent of resection Average time to progression Recurrence rate Frequency of MRI-detected recurrence
*Mixed grade tumour studies*				
Korones et al. (2001) [8] USA 1990–1999 Retrospective case series	To determine the frequency of detection of recurrent/progressive brain tumours in asymptomatic children are detected by surveillance MRI scans, and to compare the survival of children with asymptomatic recurrence compared to those whose recurrences are detected by symptoms	**Included**: pts with brain tumour aged < 21 at diagnosis and for which neuro-imaging surveillance was performed exclusively by MRI **Excluded**: pts with spinal cord tumours or children followed by CT scans **N** = 112 [although paper focuses exclusively on the 46 recurrent pts (67% male)] **Median age at diagnosis**: (n = 46) 6.5 years (0.25–21) **Average follow-up**: NR **Tumour type**: both low- and high-grade tumours, including 13 (28%) low-grade tumours pts Astrocytoma (WHO grade unspecified) n = 11 (24%) Ganglioglioma: n = 2 (4%) **Tumour location**: NR **Previous treatments**: NR	Surveillance MRI: details of magnet and image sequences NR **Average frequency of imaging**: 1 scan every 5.3 months (range 1/2.3 to 1/11.8 months) **Average number of images per pt**: NR for low-grade tumour pts only	Median time to recurrence by tumour grade Recurrence rate by symptomatic status Median OS by symptomatic status at recurrence 2-year OS from time of recurrence by symptomatic status at recurrence

*asymp* asymptomatic, *ChemT* chemotherapy, *CT* computed tomography, *DFA* diffuse fibrillary astrocytoma, *FLAIR* fluid-attenuated inversion recovery, *Gd* gadolinium, *GTR* gross total resection, *LGG* low grade glioma, *N* number of pts in study, *NF1* neurofibromatosis type 1, *NR* not reported, *OS* overall survival, *PA* pilocytic astrocytoma, *PFS* progression-free survival, *pt(s)* patient(s), *RFS* recurrence-free survival, *RT* radiotherapy, *STR* sub-total resection, *symp* symptomatic, *WHO* World Health Organisation, ± with or without

The six low-grade tumour studies included patients with the following tumour types: pilocytic astrocytoma (n = 297), ganglioglioma (n = 22), dysembryoplastic neuroepithelial tumour (DNET) (n = 6), glioneuronal tumours (n = 1), non-pilocytic astrocytoma/diffuse fibrillary astrocytoma (DFA) (n = 18), optic glioma (n = 7), oligodendroglioma (n = 3) and ‘other’ astrocytoma (n = 3).

### Korones [8]

This was the only mixed grade tumour study but it also differed from the other studies in that it did not provide a breakdown of patients at the beginning of the study, instead reporting only the number of recurrences by tumour type. Thirteen LGG patients recurred (11 astrocytomas and two gangliogliomas). However, as the number of non-recurrent LGG patients was not reported, recurrence rates were not calculable and therefore data from this study is not comparable with that from the other studies. Of the 13 recurrences, ten patients (nine astrocytoma and one ganglioglioma) were asymptomatic at recurrence giving a diagnostic yield of scanning of 3.7% (or one recurrence detected every 27 scans). Recurrent patients were scanned with a frequency of one scan every 5.3 months. Median time to recurrence was 2.3 years and OS was 100%. Neither outcome was reported by tumour type.

### MRI protocols

Four studies reported details regarding MRI scanners used, image sequences, weighting and contrast enhancement [9, 11, 13, 14]. (For details, see Table 1).

### Imaging schedules/frequency of imaging

Whilst the reporting of the frequency of scanning varied between studies, with some [9–11] reporting scanning intervals and others [9, 11, 12] reporting the number of scans per patient, a discernible pattern emerged whereby imaging was at its most frequent within the first year (2–4 follow-up scans), reducing to around half this frequency in year two and then becoming annual thereafter for the duration of follow-up. Vassilyadi et al. [14] reported frequency in terms of scanning intervals by extent of resection, with STR patients undergoing almost twice as many scans as GTR patients (one scan every 6 months vs 11 months respectively) while Udaka et al. [13] reported intervals with respect to recurrent patients only (1 scan every 3.5 months) rather than the total number of patients at the beginning of the study.

Five studies reported the average number of MRI scans per patient for the duration of follow-up [9, 11–14], ranging from six [11] to 17 scans [13]. This however is somewhat misleading as studies were inconsistent in terms of reporting as Udaka et al. [13] reported the number of scans for recurrent patients only, Alford et al. [9] the average number of scans for GTR patients (median nine) and Dodgshun et al. [10] stated the recommendations pertaining at their institution at the time of publication (nine scans in the first 5 years).

Vassilyadi et al. [14] reported the average number of scans by tumour sub-group with the median for PA patients comparable to that for non-PA patients (seven vs eight/nine respectively). The distribution of scans over the follow-up period was not reported.

### Rates of recurrence

Overall, of the total of 357 patients, 98 cases (24%) of recurrence occurred. Recurrence rates by study ranged from 5 to 41% of patients. Five studies reported recurrence by symptomatic status at recurrence, of which 0–35% of patients were symptomatic while 65–100% of patients were asymptomatic [10–14]. (See Table 2).

**Table 2:** Frequency of scanning, recurrence rates and timing of recurrences

Study [ref]	N (GTR)	Average follow-up years (range)	Average number of MRI scans by years						Rate of recurrence/progression: n (%)			Median time to recurrence/progression years (range)	Median time to recurrence years (range)		Timing of recurrence (years post-primary treatment) N (%)					
			Year 1	Year 2	Year 3	Year 4	Year 5	Years 1–5	Total	Symp	Asymp		Symp	Asymp	Year 1	Year 2	Year 3	Year 4	Year 5	> 5 years
*Low grade tumour studies*																				
Alford et al. [9]	53 (41)	6.1 (2.1–12.3)	4	4	2	1	1	12	10 (19)	NR	NR	GTR pts (n = 6): 0.64 (0.26–6.42) STR pts (n = 4): 0.42 (range NR)	NR	NR	NR	NR	NR	NR	NR	NR
Dodgshun et al. [10]^a^	67 (67)	NR	NR	NR	NR	NR	NR	12*	3 (5)	0(0)	3 (100)	1.9 (0.75–2.75)	N/A	1.9 (07.5 − 2.75)	1 (33)	–	2 (67)	–	–	–
Dorward et al. [11]	40 (40)	5.6 (2.1–9.8)	2	1	1	1	1	6	11 (28)	1 (9)	10 (91)	0.53 (0.17–4.02)	NR	NR	10 (91)	–	–	1 (9)	–	–
Kim et al. [12]	67 (67)	6.6 (1.0–14.7)	4	2	1	1	1	9	13 (19)	0 (0)	13 (100)	1.0 (0.24–10.71)	N/A	1 (0.24–10.71)	7 (54)	–	1 (8)	1 (8)	2 (15)	2 (15)
Udaka et al. [13]^b^	102 (38)	NR	3.4	3.4	3.4	3.4	3.4	17	46 (41) GTR:9 STR:35	16 (35)	30 (65)	2.28 (0.17–11)^ϒ^	NR	NR	21 (48)	9 (20)	8 (18)	–	–	6 (14)
Vassilyadi et al. [14]	28 (19)	PA: 6.0 (NR) Non-PA: 7.0 (NR)	NR	NR	NR	NR	NR	7–8	2 (7) STR	0 (0)	2 (100) STR	0.33 (0.25–0.42)	0	0.33 (0.25–0.42)	2 (100)	–	–	–	–	–
	357								98 (27)	20 (23)	68 (77)				41 (56)	9 (12)	11 (16)	2 (3)	2 (3)	8 (10)
*Mixed grade tumour studies*																				
Korones et al. [8]^c^	NR	NR	2	2	2	2	2	10	13	3 (23)	10 (77)	2.33 (0.58–4.08)	NR	NR	NR	NR	NR	NR	NR	NR

*Asymp*. asymptomatic, *GTR* gross total resection, *N/A* not applicable, *N* number of patients in study, *Non-PA* non-pilocytic astrocytoma, *nr* not reached, *NR* not reported, *PA* pilocytic astrocytoma, *STR* sub-total resection, *Symp*. symptomatic, *ϒ* mean reported rather than median

^a^For Dodgshun, the mean number of scans in years 1–5 (i.e. 12 scans (7–20)) is based on a sample of 33 pts with at least 5-years follow-up from diagnosis

^b^For Udaka, reporting of timing is based on patients with known extent of resection (i.e. 44/46 pts)

^c^Korones is a mixed tumour study (n = 112) with a total of 46 recurrent pts, 13 of which were low-grade tumour recurrences; however the breakdown between pts with high and low-grade tumours at the beginning of the study is not reported

### Recurrence rates by tumour type

Of 297 patients with PA, 70 (24%) recurred with recurrence rates across the six studies ranging from 5% [10] to 47% [13]. Of 22 ganglioglioma [12, 13], six DNET [12] and 13 non-PA/DFA [14] patients, five, one and one patients recurred respectively. Udaka, the only study to report both first and subsequent recurrences, reported eight recurrences across three unspecified PA patients [13]. (See Table 3).

**Table 3:** Recurrence rates and timing of recurrence by tumour type

Study [ref]	N of pts	Patients with recurrent/progressive disease: n (%)			Median time to recurrence: years (range)	Median time to recurrence: years (range)	
		Total	Symp	Asympt		Sympt	Asympt
*(a) Low-grade tumour studies*							
Pilocytic astrocytoma							
Alford et al. [9]	53	10 (19)	NR	NR	GTR pts (n = 6) 0.64 (0.26–6.42) STR pts (n = 4) 5.23 (range NR)	NR	NR
Dodgshun et al. [10]	67	3 (5)	0 (0)	3 (100)	1.9 (0.75–2.75)	0	1.9 (07.5–2.75)
Dorward et al. [11]	40	11 (28)	1 (9)	10 (91)	0.53 (0.17–4.02)	NR	NR
Kim et al. [12]	46	9 (20)	0 (0)	9 (100)	NR	NR	NR
Udaka et al. [13]	76	36 (47)	NR	NR	NR	NR	NR
Vassilyadi et al. [14]	15	1 (7)	0 (0)	1 (100)	0.25	0	0.25
Totals	297	70 (24)	1 (4)	23 (96)			
Diffuse fibrillary astrocytoma							
Vassilyadi et al. [14]	13	1 (8)	0 (0)	1 (100)	0.42	0	0.42
Other astrocytoma (WHO grade not specified)							
Udaka et al. [13]	3	8 (267)	NR	NR	NR	NR	NR
Ganglioglioma							
Kim et al. [12]	14	3 (21)	0	3 (100)	NR	NR	NR
Udaka et al. [13]	8	2 (25)	NR	NR	NR	NR	NR
Totals	22	5 (23)	0	3 (100)			
Dysembryoplastic neuroepithelial tumours (DNET)							
Kim et al. [12]	6	1 (17)	0	1 (100)	NR	NR	NR
*(b) Mixed-grade tumour study (Korones)*							
Other astrocytoma (WHO grade not specified)		11^a^	2 (18)	9 (82)	NR	NR	NR
Ganglioglioma		2	1 (50)	1 (50)	NR	NR	NR

*Asymp*. asymptomatic, *GTR* gross total resection, *N* number of patients in study, *NR* not reported, *STR* sub-total resection, *Symp*. symptomatic

^a^Korones et al. [8] was the only study which did not provide a breakdown of the patients at the beginning of the study in terms of tumour type and, as such, the number of recurrences in this study (n = 13) has not been taken into account when calculating the percentage of the total number of patients at baseline with each tumour type which went on to experience a recurrence

Asymptomatic recurrence rates for PA were calculable in four studies, ranging from 82 to 100% [10–12, 14]. Across the four studies, 96% of recurrences were asymptomatic. Kim reported that the three recurrences of ganglioglioma and one recurrence of DNET were all asymptomatic at recurrence [12]. The patient with progressive non-PA was asymptomatic at recurrence [14].

There were no recurrences observed in patients with glioneuronal tumour, optic glioma and oligodendroglioma although the number of patients with each of these tumour types was so small that no inferences should be drawn regarding recurrence in these patients.

### Recurrence rates by tumour site

While most studies reported patients by both tumour type and location, most outcomes, including recurrence, were reported by tumour type alone and therefore it was not possible to discern the effect of tumour location on recurrence. Only Dodgshun et al. [10], with nine of 67 PAs located supratentorially, reported that there was ‘no difference in recurrence rate with regard to tumour site (p = 0.37).’

### Recurrence rates by extent of resection

The three studies consisting solely of GTR patients reported recurrence rates of 4% (n = 3/67), 28% (n = 11/40) and 19% (n = 13/67) respectively [10–12]. Three of the four studies with both GTR and STR patients reported recurrence rates for GTR patients of 15% (n = 6/41), 24% (n = 9/38) and 0% (n = 0/19) respectively and rates of recurrence for STR patients of 33% (n = 4/12), 55% (n = 35/64) and 22% (n = 2/9) respectively [9, 13, 14] (See Table 4).

**Table 4:** Breakdown of low-grade tumour patients by extent of resection

Author (year) [ref]	N	Rec N	GTR n (%)		STR n (%)		N/A
			All pts	Rec pts	All pts	Rec pts	
*GTR pts only*							
Dodgshun et al. (2016) [10]	67	3	67 (100)	3 (4)	N/A	N/A	N/A
Dorward et al. (2010) [11]	40	11	40 (100)	11 (28)	N/A	N/A	N/A
Kim et al. (2014) [12]	67	13	67 (100)	13 (19)	N/A	N/A	N/A
*GTR and STR pts*							
Alford et al. (2016) [9]	53	10	41^a^ (77)	6 (15)	12 (23)	4 (33)	N/A
Korones et al. (2014) [8]	NR	–	NR	NR	NR	NR	NR
Udaka et al. (2013) [13]	102	46	38 (37)	9 (24)	64 (63)	35 (55)	2 (4)
Vassilyadi et al. (2009) [14]	28	2	19 (68)	0 (0)	9 (32)	2 (22)	N/A
Totals	357	85	272 (76)	42 (15)	85 (24)	41 (48)	2 (3)

*GTR* gross total resection, *N/A* not applicable, *Pts* patients, *N* number of patients in study, *N* total number of patients in study, *NR* not reported, *NR* not reported, *Rec n* number of recurrent patients, *STR* sub-total resection

^a^GTR/indeterminate

### Diagnostic yield of imaging

The diagnostic yield of MRI, or the number of scans identifying recurrence as a proportion of total scans, was reported in three studies [9, 10, 14]. For Alford, diagnostic yield was 2% (i.e. one recurrence detected every 50 scans) based on 6 of 41 predominantly GTR patients [9]. As the symptomatic status of recurrent patients was not reported, diagnostic yield by symptomatic status was not calculable. For Dodgshun, diagnostic yield was 0.25% (one asymptomatic recurrence detected with 399 scans) based on 33 patients with at least 5 years follow-up [10]. For Vassilyadi, diagnostic yield was 1% (two asymptomatic recurrences detected with 216 scans) in STR patients [14].

### Time to recurrence

Five studies [8, 10–14] reported average time to recurrence post-primary treatment ranging from 0.33 [14] to 2.33 years [8]. (See Table 2). In two studies, recurrence was 100% asymptomatic with median times to recurrence of 1.9 and 1.0 years respectively [10, 12]. Neither of the studies containing mixed low-grade tumour types reported median time to recurrence by tumour type [12, 13].

### Time to recurrence by extent of resection

Three studies with exclusively GTR patients reported median times to recurrence of 0.53 [11], 1.0 [12] and 1.9 [10] years respectively. Of the three studies containing both GTR and STR patients, two reported median times to recurrence of 2.28 [13] and 0.33 years [14] respectively. Only Alford et al. [9] reported median time to recurrence solely by extent of resection [GTR 0.64 vs STR 0.42 years respectively (p < 0.0001)].

### Timing of recurrences

Five studies provided data on the timing of recurrences post-diagnosis/primary treatment [10–14]. Collectively, 56% of recurrences took place within the first year post-primary treatment (with 46% of these within the first six months), 68% by year two and 90% by year five. Neither of the studies with mixed tumour populations reported timing of recurrences by tumour type [12, 13].

### Patient management post-recurrence

Four studies reported details of patient management following recurrence with respect to 29 patients, 28 of whom were asymptomatic [10–12, 14]. Twenty-six patients underwent a change in their management including repeat surgery (n = 21), chemotherapy (n = 2), radiotherapy (n = 2) and radiosurgery (n = 1). The remaining three patients were observed.

### Survival

All but two studies reported some type of survival outcome [9, 14].

### Overall survival (OS)

Four studies reported OS up until the time of reporting [10–13]. In two studies of PA [10, 11] OS was100% and in the two mixed tumour studies [12, 13] OS was 96 and 100% respectively, all measured from recurrence.

### Surrogate survival outcomes

Four studies reported surrogate survival outcomes [10–13]. Dodgshun et al. [10] reported 5-year EFS of 95% (95% CI 90–100%). Dorward reported RFS by evidence of ‘nodular enhancement’ on surveillance MRI within the first 3–6 months, with both the 5- and 10-year RFS for PA patients whose scans lacked evidence of nodular enhancement of 90% compared to patients whose scans evidenced nodular enhancement of 44 and 22% respectively [11]. Kim reported 2- and 5-year RFS for 67 patients of 90% and 82% respectively, as well as RFS by tumour type, with 2- and 5-year RFS for PA, ganglioglioma and DNET of 87 and 82, 93 and 85 and 100 and 67% respectively [12].

Udaka reported median PFS for all 102 patients (4.7 years) and separately for patients with PA (4.2 years) who represented 75% of the study population [13]. Median PFS for GTR patients was significantly greater than STR/biopsy patients [not reached versus 2.1 years respectively (p = 0.012)]. Udaka also found that while recurrence was reduced in GTR patients, it occurred earlier compared to those with less than total resection (16.6 vs 25.8 months, respectively).

### Quality of survival

None of the studies reported quality of survival of the children and their families.

## Discussion

This systematic review was borne out of discussions between the project team and the PPI group, which consisted largely of mothers of children with CNS tumours. Of particular interest to the PPI group was surveillance scanning and its frequency. They remarked that scanning was a significant and time-consuming part of their lives and a major source of anxiety to the whole family before, during and after each scan. However, they were unanimous that scanning was something they were prepared to endure so long as this was an evidence-based practice that ultimately benefitted their children. They were surprised to learn that, despite being routine practice, there are no internationally adopted guidelines for the frequency and duration of surveillance MRI in paediatric CNS tumours. This issue is not only important to patients and their families but also to health care systems such as the NHS in terms of direct and indirect healthcare costs. A surveillance imaging programme needs to detect recurrent disease ahead of onset of signs/symptoms and to result in changes in patient management which bestow a long-term clinical benefit in terms of improved patient outcomes (i.e. reduced mortality and/or improved quality of survival). Ultimately, the assessment of both the benefits and costs of this practice should be based on research evidence and this is what prompted us to undertake the current review.

Six low-grade tumour surveillance imaging studies were excluded from the review as they employed both CT and MRI as surveillance imaging modalities but did not report results separately by modality [15–20]. No comparative studies assessing the effectiveness of routine surveillance screening with MRI were identified. The evidence base to guide the clinical practice of surveillance MRI for children with low-grade CNS tumours consisted of seven small retrospective, single arm observational studies in which data—acquired for clinical purposes rather than assessment of surveillance imaging protocols—was analysed to determine the optimal frequency and/or duration of surveillance MRI. Six of these studies consisted solely of patients (n = 357) with low-grade CNS tumours while one study, comprising a mixture of low- and high-grade tumour patients (n = 112), reported on 13 low-grade tumour patients [8]. In all studies, MRI was employed exclusively as the imaging modality with all of the patients having undergone surgery as a primary treatment, achieving either GTR or STR. For all studies, both the number and rates of recurrence were low, with the majority of recurrences diagnosed asymptomatically via surveillance MRI and tending to occur within the first 2 years following primary treatment, suggesting there may be scope for reducing the number and frequency of later scans (10% of recurrences occurred post-five years, although patient characteristics of these individuals were not described). The extent of initial resection also appeared to be associated with recurrence, with patients achieving GTR significantly less likely to experience recurrence.

Although all seven studies reported essentially similar results, study authors differed in their interpretation, leading to opposing conclusions regarding the optimal frequency and/or duration of surveillance with respect to GTR patients (see Online Resource 6). For example, Alford et al. [9] concluded that frequent imaging of GTR patients may be unwarranted beyond the radiological confirmation of GTR documented on two consecutive scans separated by at least 3 months; likewise the study by Vassilyadi et al. [14] concluded that GTR patients may not benefit from surveillance, although this was based solely on recurrences in two STR patients. Conversely, Udaka et al. [13] advised caution, recommending surveillance up to 5 years, irrespective of the extent of resection. Similarly Dorward, despite identifying associations predictive of recurrence in GTR patients (p < 0.05), also erred on the side of caution, albeit based on limited data (i.e. one delayed recurrence) [11]. Both Dodgshun et al. [10] and Kim et al. [12], based on the timing of recurrences post-diagnosis, suggested reduced imaging schedules but argued that long-term imaging (5 and 10 years respectively) for GTR patients was necessary although, again, this was based on a very small number of recurrences—three and 13 recurrences respectively. Overall, it is interesting to note that all of these conclusions and recommendations were based on low recurrence numbers, ranging from 0 [14] to 13 [12].

As demonstrated by the study authors, drawing conclusions from these studies is problematic. The potential for bias with case series studies is considerable making any conclusions from this review highly tentative and to be viewed with extreme caution. For instance, patient populations across the studies were highly selected with the main patient group being children with posterior fossa PA that had been completely resected. Half the studies excluded patients with low-grade tumour predisposition syndromes such as NF1 and tuberous sclerosis [9, 11, 13]. One study [13] differed from the others by including a large number of patients with low grade gliomas at all sites who had immediate adjuvant therapy post-surgery, making them a population with a significantly higher risk of recurrence. All of this selection bias is likely to skew the results of this review. The review question needs to be properly investigated within an RCT. Of particular importance in paediatric low-grade tumour studies, where survival is generally excellent, patient-reported quality of survival should be a priority: none of the studies reported this outcome. Future trials should also examine potential adverse events resulting from the repeated administration of contrast materials (e.g. Gadolinium [21]) and in younger children, anaesthesia and sedatives. Although an RCT would be challenging to design and conduct, the results of this review demonstrate that we are at equipoise as to the optimum scanning regimen. Scanning is a vital part of the treatment pathway for children with CNS tumours and has the potential to improve survival but also has risks associated with it. Its optimum use therefore needs to be established.

To conclude, despite the existence of various consensus recommendations [22, 23], this systematic review did not identify any studies that were able to inform best practice as to the frequency or duration of surveillance MRI in asymptomatic children with LGG. The findings could however inform the development of future clinical trials, particularly regarding scanning frequency and duration.

## Electronic supplementary material

Below is the link to the electronic supplementary material.

Online Resource 1 Medline Search Strategy. Supplementary material 1 (PDF 90 KB)

Online Resource 2 List of excluded studies. Supplementary material 2 (PDF 111 KB)

Online Resource 3 Data extraction and quality assessment proforma. Supplementary material 3 (PDF 113 KB)

Online Resource 4 Definitions of extent of resection and recurrence provided by authors. Supplementary material 4 (PDF 174 KB)

Online Resource 5 Quality appraisal of included primary studies. Supplementary material 5 (PDF 171 KB)

Online Resource 6 Study authors’ conclusions. Supplementary material 6 (PDF 224 KB)
