# Supplementary material for: The utility of routine surveillance screening with magnetic resonance imaging (MRI) to detect tumour recurrence in children with low-grade central nervous system (CNS) tumours: a systematic review
Source: J Neurooncol. 2018 Jun 9;139(3):507–22. doi: 10.1007/s11060-018-2901-x (PMC6132973; doi:10.1007/s11060-018-2901-x)
Supplement: Supplementary file 1 — Medline Search Strategy. Supplementary material 1 (PDF 90 KB) [file 11060_2018_2901_MOESM1_ESM.pdf]

Online Resource 1: Search strategy

Searches for published studies were undertaken using the following databases: MEDLINE (OvidSP); MEDLINE In-Process Citations & Daily Update (OvidSP); EMBASE (OvidSP); Cochrane Database of Systematic Reviews (CDSR)(Wiley); Cochrane Central Register of Controlled Trials (CENTRAL)(Wiley); CINAHL Plus (EBSCO); DARE (CRD website); HTA (CRD website).

The search strategy for Medline (OvidSP) 1985 - January week 1, 2017, is as follows:

1. Glioma/ or Brain Neoplasms/ or Meningioma/ or Glioblastoma/ or Astrocytoma/
2. ((brain or brainstem or intracranial or posterior fossa) adj3 (cancer* or carcinom* or tumor* or tumour* or neoplasm*)).mp.
3. (Astrocytoma* or Brain Stem Glioma* or Medulloblastoma*or Primitive Neuroectodermal Tumo?r* or ganglioneuroblastoma* or CNS neuroblastoma* or Ependymoblastoma or Medulloepithelioma or Pineal Parenchymal Tumour* or (Atypical Teratoid adj1 tumo?r*) or Oligoastrocytoma or ((Pilocytic or Gemistocytic) adj1 astrocytoma*) or ependymoma or primitive neuroectal tumo?r*).mp.
4. (((Diffuse fibrillary or Gemistocytic or Pilocytic Pilomyxoid Protoplasmic Subependymal giant cell) adj1 astrocytoma*) or Oligoastrocytoma or Oligodendroglioma or Oligoastrocytoma or Pleomorphic xanthoastrocytoma or ((astrocytoma or oligoastrocytoma or oligodendroglioma) adj1 astrocytoma*) or Glioblastoma or Gliomatosis cerebri or Gliosarcoma or ((diffuse intrinsic pontine glioma or low grade brain stem) adj1 glioma) or ((classic or desmoplastic or nodular or large cell or nodularity) adj1 medulloblastoma*) or Primitive Neuroectodermal Tumo?r* or ((ganglioneuroblastoma or neuroblastoma) adj 1central nervous system*) or Ependymoblastoma or Pineoblastoma or pineal parenchymal tumo?r* or (central nervous system adj1 atypical teratoid) or (central nervous system adj 1 rhabdoid tumo?r*) or Germinomas or ((immature or mature or malignant transformation) adj2 teratomas)).mp.
5. 1 or 2 or 3 or 4
6. exp Surgical Procedures, Operative/
7. surg*.mp.
8. debulk*.mp.
9. cytoreduc*.mp.
10. 6 or 7 or 8 or 9
11. (chemotherap* or antineoplastic agents or cytotoxic or alkylating agents or nitrosoureas or antimetabolite* or antitumor?r or ((antibod* or monoclonal) adj 3 Human*) or plant alkyloid* or (hormone* adj 1 agent*) or anthracycline* * or systemic therap*).mp.
12. (Everolimus or Afinitor or Cetuximab or Erbitux or Bevacizumab or Avastin or Cediranib or Recentin or lomustine or CCNU or CeeNU or carmustine or BiCNU or Carustine or Ethylnitrosourea or Streptozocin or Sorafenib or Nexavar or tipifarnib or Zarnestra or Erlotinib or Tarceva or Sorafenib or Nexavar or temsirolimus or Torisel or Sunitinib or Sutent or irinotecan or Camptosar or Campto or Vandetanib or Caprelsa or Cabozantinib or Cometriq or XL184 or Axitinib or AG013736 or Inlyta
).mp.
13. 11 or 12
14. exp Immunotherapy/ae, cl, ct, mt, mo, nu, px, st
15. exp Genetic Therapy/ae, cl, ct, mt, mo, nu, ut
16. exp Imaging, Three-Dimensional/ or exp Whole Body Imaging/ or exp Magnetic Resonance Imaging/
17. exp Tomography, Emission-Computed/ or exp Four-Dimensional Computed Tomography/ or exp Tomography/ or exp Tomography, Emission-Computed, Single-Photon/ or exp Positron-Emission Tomography/
18. 16 or 17
19. (radiation therapy or radiotherap* or intensity modulat* radiotherapy*or radiosurgery or radiation oncology or reduced boost volume radiotherap* or hyper fractionat* stereotactic radiotherap*or adjuvant radiotherap* or body radiotherap* stereotactic*or computer assisted radiotherap*or computer assisted radiotherap*planning or conformal radiotherap* or dosage* radiotherap* or dose fractionation* radiotherap* or high energy radiotherap*or implant radiotherap*or intensity or modulated radiotherap*or interstitial radiotherap*orimage guided radiotherap*or stereotactic*guid* radiotherap* or local therap* or proton therap* or proton adj2 therap* or proton beam therap* or proton adj2 radiation or proton radiation therap* or proton adj2 radiotherap* or proton adj2 irradia* or PBT).mp.
20. 10 or 13 or 14 or 15 or 18 or 19
21. 5 and 20
22. (Response or overall survival or progression* free survival or event* free survival or time to recurrence or time to progression or disease* free interval* or endocrinopath* or ((growth or thyroid) adj 1 hormone adj 3 deficienc*) or ((glucocorticoid or gonadotropin) adj 3 deficienc*) or endocrine dysfuct* or (cardiac function*

adj 3 impair*) or ataxia or spastic paresis or visual dysfunction or epilepsy or hemiparesis or neurolog* deficit*).mp.
23. 21 and 22
24. limit 23 to (yr="1985 -Current" and ("newborn infant (birth to 1 month)" or "infant (1 to 23 months)" or "preschool child (2 to 5 years)" or "child (6 to 12 years)" or "adolescent (13 to 18 years)" or "young adult (19 to 24 years)") and humans)

The utility of routine surveillance screening with magnetic resonance imaging (MRI) to detect tumour recurrence in children with low grade central nervous system (CNS) tumours: a systematic review

Journal of Neuro-oncology

Simon P. Stevens,[1] Caroline Main,[1] Simon Bailey,[2] Barry Pizer,[3] Martin English,[5] Robert Phillips,[6] Andrew Peet,[4] Shivaram Avula,[3] Sophie Wilne,[7] Keith Wheatley,[1] Pamela R. Kearns,[1,5] Jayne S.Wilson[1]

[1] Cancer Research UK Clinical Trials Unit (CRCTU), Institute of Cancer and Genomic Sciences, University of Birmingham, UK
[2] Sir James Spence Institute of Child Health, Royal Victoria Infirmary, Newcastle-Upon-Tyne
[3] Alder Hey Children's NHS Foundation Trust, Liverpool, UK
[4] Institute of Cancer and Genomic Sciences, University of Birmingham, UK
[5] Birmingham Women and Children's Hospital NHS Foundation Trust, Birmingham, UK
[6] Centre for Reviews and Dissemination (CRD), University of York, UK
[7] Queen's Medical Centre, Nottingham University Hospitals' NHS Trust, Nottingham, UK

Correspondence:
Jayne Wilson
UK; Tel: +441214149273
Email: j.s.wilson.1@bham.ac.uk
