# Supplementary material for: The utility of routine surveillance screening with magnetic resonance imaging (MRI) to detect tumour recurrence in children with low-grade central nervous system (CNS) tumours: a systematic review
Source: J Neurooncol. 2018 Jun 9;139(3):507–22. doi: 10.1007/s11060-018-2901-x (PMC6132973; doi:10.1007/s11060-018-2901-x)
Supplement: Supplementary file 2 — List of excluded studies. Supplementary material 2 (PDF 111 KB) [file 11060_2018_2901_MOESM2_ESM.pdf]

Online Resource 2: List of excluded studies

Study details (author(s), title and publication details)	Reason for exclusion
Antony R, Wong KE, Patel M, Olch AJ, McComb G, Krieger M, et al. A retrospective analysis of recurrent intracranial ependymoma. Pediatric Blood & Cancer. 2014;61(7):1195-201.	Not a surveillance neuroimaging study.
Elbabaa S. Complication avoidance during resection of fourth ventricular brain tumors with brain stem invasion: Value of intra-operative emg mapping of facial colliculus in preservation of facial nerve function in pediatric patients. Child's Nervous System. 2012;28 (9):1658.	Not a surveillance neuroimaging study.
Good CD, Wade AM, Hayward RD, Phipps KP, Michalski AJ, Harkness WF, et al. Surveillance neuroimaging in childhood intracranial ependymomas: how effective, how often, and for how long? Journal of Neurosurgery. 2001;94(1):27-32.	Both CT and MRI employed at different stages of the study, with results not reported separately by neuroimaging modality.
Gunther J, Sato M, Chintagumpala M, Jo E, Paulino A, Adesina A, et al. Imaging changes in pediatric patients treated with proton beam radiation therapy compared to intensity modulated radiation therapy for intracranial ependymoma. Neuro-Oncology. 2014;16:i120.	Not a surveillance neuroimaging study.
Gunther JR, Sato M, Chintagumpala M, Jo E, Paulino A, Adesina A, et al. Comparison of imaging changes in pediatric patients treated with proton beam radiation therapy versus intensity modulated radiation therapy for intra-cranial ependymoma. International Journal of Radiation Oncology Biology Physics. 2014;1):S115.	Not a surveillance neuroimaging study.
Gururangan S, Hwang E, Herndon IJE, Fuchs H, George T, Coleman RE. [18F]fluorodeoxyglucose-positron emission tomography in patients with medulloblastoma. Neurosurgery. 2004;55(6):1280-8.	Not a surveillance neuroimaging study.
Hall WA, Martin AJ, Liu H, Pozza CH, Casey SO, Michel E, et al. High-field strength interventional magnetic resonance imaging for pediatric neurosurgery. Pediatric Neurosurgery. 1998;29(5):253-9.	Not a surveillance neuroimaging study.
Hirpara DH, Bhatt MD, Katrin S (2016) Utility of Long-Term Surveillance Neuroimaging Five Years Post-Diagnosis in the Management of Pediatric Brain Tumours. Austin Pediatr Oncol 1 (1): 1002.[5]	Both CT and MRI employed at different stages of the study, with results not reported separately by neuroimaging modality.
Kornreich L, Schwarz M, Karmazyn B, Cohen IJ, Shuper A, Michovitz S, et al. Role of MRI in the management of children with diffuse pontine tumors: a study of 15 patients and review of the literature. Pediatric Radiology. 2005;35(9):872-9.	Surveillance MRI study with high-grade tumour patients only
Kovanlikaya A, Karabay N, Çakmakçi H, Uysal K, Olgun N, Ergör G. Surveillance imaging and cost effectivity in pediatric brain tumors. European Journal of Radiology. 2003;47(3):188-92.[3]	Both CT and MRI employed at different stages of the study, with results not reported separately by neuroimaging modality.
Kruer MC, Kaplan AM, Etzl MM, Jr., Carpentieri DF, Dickman PS, Chen K, et al. The value of positron emission tomography and proliferation index in predicting progression in low-grade astrocytomas of childhood. Journal of Neuro-Oncology. 2009;95(2):239-45.	Not a surveillance neuroimaging study.
Laprie A, Pirzkall A, Haas-Kogan DA, Cha S, Banerjee A, Le TP, et al. Longitudinal multivoxel MR spectroscopy study of pediatric diffuse brainstem gliomas treated with radiotherapy. International Journal of Radiation Oncology, Biology, Physics. 2005;62(1):20-31.	Not a surveillance neuroimaging study.
Lechon FC, Fowkes L, Khabra K, Martin-Retortillo LM, Marshall LV, Schrey D, et al. Characterisation of pseudoprogression in children and adolescents with diffuse intrinsic pontine gliomas. Neuro-Oncology. 2014;16:i46.	Not a surveillance neuroimaging study.
Mendel E, Levy ML, Raffel C, McComb JG, Pikus H, Nelson MDJ, et al. Surveillance Imaging in Children with Primitive Neuroectodermal Tumors. Neurosurgery. 1996;38(4):692-5.	Both CT and MRI employed at different stages of the study, with results not reported separately by neuroimaging modality.
Minn AY, Pollock BH, Garzarella L, Dahl GV, Kun LE, Ducore JM, et al.	Both CT and MRI employed at

Study details (author(s), title and publication details)	Reason for exclusion
Surveillance neuroimaging to detect relapse in childhood brain tumors: a Pediatric Oncology Group study. Journal of Clinical Oncology. 2001;19(21):4135-40.	different stages of the study, with results not reported separately by neuroimaging modality.
Norfray JF, Tomita T, Byrd SE, Ross BD, Berger PA, Miller RS. Clinical impact of MR spectroscopy when MR imaging is indeterminate for pediatric brain tumors. AJR American Journal of Roentgenology. 1999;173(1):119-25.	Not a surveillance neuroimaging study.
Ogiwara H, Bowman RM, Tomita T. Long-term follow-up of pediatric benign cerebellar astrocytomas. Neurosurgery. 2012;70(1):40-7.[6]	Both CT and MRI employed at different stages of the study, with results not reported separately by neuroimaging modality.
Packer RJ, Zimmerman RA, Bilanuik LT, Leurssen TG, Sutton LN, Bruce DA, et al. Magnetic resonance imaging of lesions of the posterior fossa and upper cervical cord in childhood. Pediatrics. 1985;76(1):84-90.	Not a surveillance neuroimaging study.
Parizek J, Mericka P, Nemecek S, Nemeckova J, Spacek J, Suba P, et al. Posterior cranial fossa surgery in 454 children. Comparison of results obtained in pre-CT and CT era and after various types of management of dura mater. Childs Nervous System. 1998;14(9):426-38; discussion 39.	Not a surveillance neuroimaging study.
Perreault S, Lober RM, Carret AS, Zhang G, Hershon L, Decarie JC, et al. Surveillance imaging in children with malignant CNS tumors: low yield of spine MRI. J Neurooncol. 2014;116(3):617-23.	Surveillance MRI study with high-grade tumour patients only
Phi JH, Cho BK, Kim SK, Paeng JC, Kim IO, Kim IH, et al. Germinomas in the basal ganglia: magnetic resonance imaging classification and the prognosis. Journal of Neuro-Oncology. 2010;99(2):227-36.	Not a surveillance neuroimaging study.
Rodriguez Gutierrez D, Manita M, Jaspan T, Dineen RA, Grundy RG, Auer DP. Serial MR diffusion to predict treatment response in high-grade pediatric brain tumors: a comparison of regional and voxel-based diffusion change metrics. Neuro-Oncology. 2013;15(8):981-9.	Not a surveillance neuroimaging study.
Roebuck JD, Villablanca GJ, Maher K, Nelson Jr. DM. Surveillance imaging in children with medulloblastoma (posterior fossa PNET). Pediatric Radiology. 2000;30(7):447-50.	Both CT and MRI employed at different stages of the study, with results not reported separately by neuroimaging modality.
Saito R, Kumabe T, Kanamori M, Sonoda Y, Watanabe M, Mugikura S, et al. Early response to chemotherapy as an indicator for the management of germinoma-like tumors of the pineal and/or suprasellar regions. Journal of Clinical Neuroscience. 2014;21(1):124-30.	Not a surveillance neuroimaging study.
Saunders DE, Hayward RD, Phipps KP, Chong WK, Wade AM. Surveillance neuroimaging of intracranial medulloblastoma in children: how effective, how often, and for how long? Journal of Neurosurgery. 2003;99(2):280-6.	Both CT and MRI employed at different stages of the study, with results not reported separately by neuroimaging modality.
Saunders DE, Phipps KP, Wade AM, Hayward RD. Surveillance imaging strategies following surgery and/or radiotherapy for childhood cerebellar low-grade astrocytoma. J Neurosurg. 2005;102(2 Suppl):172-8.[7]	Both CT and MRI employed at different stages of the study, with results not reported separately by neuroimaging modality.
Shaw DWW, Geyer JR, Berger MS, Milstein J, Lindsley KL. Asymptomatic recurrence detection with surveillance scanning in children with medulloblastomaJournal of Clinical Oncology. 1997;15(5):1811-3.	Both CT and MRI employed at different stages of the study, with results not reported separately by neuroimaging modality.
Steinbok P, Hentschel S, Cochrane DD, Kestle JR. Value of postoperative surveillance imaging in the management of children with some common brain tumors. J Neurosurg. 1996;84(5):726-32.[4]	Both CT and MRI employed at different stages of the study, with results not reported separately by neuroimaging modality.
Sutton LN, Cnaan A, Klatt L, Zhao H, Zimmerman R, Needle M, et al. Postoperative surveillance imaging in children with cerebellar astrocytomas. J Neurosurg. 1996;84(5):721-5.[8]	Both CT and MRI employed at different stages of the study, with results not reported separately by neuroimaging modality.
Torres CF, Rebsamen S, Silber JH, Sutton LN, Bilaniuk LT, Zimmerman	Both CT and MRI employed at

Study details (author(s), title and publication details)	Reason for exclusion
RA, et al. Surveillance scanning of children with medulloblastoma. New England Journal of Medicine. 1994;330(13):892-5.	different stages of the study, with results not reported separately by neuroimaging modality.
Wang Y, Zou L, Gao B. Intracranial germinoma: clinical and MRI findings in 56 patients. Childs Nervous System. 2010;26(12):1773-7.	Not a surveillance neuroimaging study.
Wechsler-Jentzsch K, Witt JH, Fitz CR, McCullough DC, Harisiadis L. Unresectable gliomas in children: tumor-volume response to radiation therapy. Radiology. 1988;169(1):237-42.	Not a surveillance neuroimaging study.
Yalcin B, Buyukpamukcu M, Akalan N, Cila A, Tezer Kutluk M, Akyuz C. Value of surveillance imaging in the management of medulloblastoma. Medical and Pediatric Oncology. 2002;38(2):91-7.	Both CT and MRI employed at different stages of the study, with results not reported separately by neuroimaging modality.
Yeom KW, Lober RM, Andre JB, Fisher PG, Barnes PD, Edwards MS, et al. Prognostic role for diffusion-weighted imaging of pediatric optic pathway glioma. Journal of Neuro-Oncology. 2013;113(3):479-83.	Not a surveillance neuroimaging study.

Key: CT: computed tomography; MRI: magnetic resonance

The utility of routine surveillance screening with magnetic resonance imaging (MRI) to detect tumour recurrence in children with low grade central nervous system (CNS) tumours: a systematic review

Journal of Neuro-oncology

Simon P. Stevens,[1] Caroline Main,[1] Simon Bailey,[2] Barry Pizer,[3] Martin English,[5] Robert Phillips,[6] Andrew Peet,[4] Shivaram Avula,[3] Sophie Wilne,[7] Keith Wheatley,[1] Pamela R. Kearns,[1,5] Jayne S.Wilson[1]

[1] Cancer Research UK Clinical Trials Unit (CRCTU), Institute of Cancer and Genomic Sciences, University of Birmingham, UK
[2] Sir James Spence Institute of Child Health, Royal Victoria Infirmary, Newcastle-Upon-Tyne
[3] Alder Hey Children's NHS Foundation Trust, Liverpool, UK
[4] Institute of Cancer and Genomic Sciences, University of Birmingham, UK
[5] Birmingham Women and Children's Hospital NHS Foundation Trust, Birmingham, UK
[6] Centre for Reviews and Dissemination (CRD), University of York, UK
[7] Queen's Medical Centre, Nottingham University Hospitals' NHS Trust, Nottingham, UK

Correspondence:
Jayne Wilson
UK; Tel: +441214149273
Email: j.s.wilson.1@bham.ac.uk
