# Supplementary material for: The utility of routine surveillance screening with magnetic resonance imaging (MRI) to detect tumour recurrence in children with low-grade central nervous system (CNS) tumours: a systematic review
Source: J Neurooncol. 2018 Jun 9;139(3):507–22. doi: 10.1007/s11060-018-2901-x (PMC6132973; doi:10.1007/s11060-018-2901-x)
Supplement: Supplementary file 3 — Data extraction and quality assessment proforma. Supplementary material 3 (PDF 113 KB) [file 11060_2018_2901_MOESM3_ESM.pdf]

Online Resource 3: Data extraction and quality assessment proforma

**Study demographics**	
Study Name / ID:	
Investigator group/study name:	
Hospital and country conducted:	
Single or multi-institutional study:	
Study title:	
Study objectives:	
Publication status of paper:	
Is the study linked to other papers? (state):	
Study design:	
Years of study:	
Overall trial N:	
**Population / Study eligibility**	
General tumour type(s):	
Tumour subtype(s) (N) :	
Tumour location:	
Tumour grade:	
Age: (median, range: mean; SD):	
Male: Female N (%)	
Inclusion criteria:	
Exclusion criteria:	
**Baseline data**	
Previous treatments:	
Response after previous treatments:	
In the case of surgery, extent of resection:	
Imaging modality used to determine post-operative status:	
**Intervention(s)**	
Intervention:	
MRI protocol and planning (including plane(s), weighting and contrast enhancement)	
Treatment intent (curative or palliative)	
Concomitant therapy:	
**Outcome definitions:-**	
Post-operative MRI scan:	
Surveillance MRI scan:	
Symptomatic:	
Asymptomatic:	
Definition of (radiographic) recurrence / progression:	
Overall survival (OS):	
Event-free survival (EFS):	
Recurrence-free survival (RFS):	
Progression-free survival (PFS):	
Measurement of tumour response:	
Short-term adverse events (toxicity)	
Long-term adverse events	
**Results**	
N in analysis:	

Average duration of follow-up (range):	
Average frequency of MRI surveillance imaging (range):	
Average number of surveillance MRI images per patient (range):	
Patients with Recurrent disease (n):	
Average age at recurrence (range):	
Average time-to-recurrence / progression (range):	
Frequency of MRI-detected recurrence / detection rate:	
Changes in patient treatment due to recurrence:	
Median survival:	
Overall survival (OS):	
Event-free survival (EFS):	
Recurrence-free survival (RFS):	
Progression-free survival (PFS):	
Tumour response rates:	
Short-term adverse events (toxicity)	
Long-term adverse events	
Quality of survival:	
Measures of family psychological functioning and anxiety:	
**Analysis**	
Methods of analysis (ITT or per protocol):	
Statistical tests used (state; comment)	
**Conclusions**	
Authors conclusions:	
Reviewers conclusions:	

**Study Quality Assessment**		**Y/N**
1.	Is the study based on a representative sample selected from the relevant population?	
2.	Are the criteria for inclusion explicit?	
3.	Were all individuals who entered the study, at a similar timepoint in their disease progression?	
4.	Was follow-up long enough for important outcomes to occur?	
5.	Were outcomes assessed using objective criteria or was blinding used?	
6.	If comparisons of sub-series are being made, was there sufficient description of the series and distribution of prognostic factors?	
Reviewer comments:		

The utility of routine surveillance screening with magnetic resonance imaging (MRI) to detect tumour recurrence in children with low grade central nervous system (CNS) tumours: a systematic review

Journal of Neuro-oncology

Simon P. Stevens,[1] Caroline Main,[1] Simon Bailey,[2] Barry Pizer,[3] Martin English,[5] Robert Phillips,[6] Andrew Peet,[4] Shivaram Avula,[3] Sophie Wilne,[7] Keith Wheatley,[1] Pamela R. Kearns,[1,5] Jayne S.Wilson[1]

[1] Cancer Research UK Clinical Trials Unit (CRCTU), Institute of Cancer and Genomic Sciences, University of Birmingham, UK
[2] Sir James Spence Institute of Child Health, Royal Victoria Infirmary, Newcastle-Upon-Tyne
[3] Alder Hey Children's NHS Foundation Trust, Liverpool, UK
[4] Institute of Cancer and Genomic Sciences, University of Birmingham, UK
[5] Birmingham Women and Children's Hospital NHS Foundation Trust, Birmingham, UK
[6] Centre for Reviews and Dissemination (CRD), University of York, UK
[7] Queen's Medical Centre, Nottingham University Hospitals' NHS Trust, Nottingham, UK

Correspondence:
Jayne Wilson
UK; Tel: +441214149273
Email: j.s.wilson.1@bham.ac.uk
