# Supplementary material for: The utility of routine surveillance screening with magnetic resonance imaging (MRI) to detect tumour recurrence in children with low-grade central nervous system (CNS) tumours: a systematic review
Source: J Neurooncol. 2018 Jun 9;139(3):507–22. doi: 10.1007/s11060-018-2901-x (PMC6132973; doi:10.1007/s11060-018-2901-x)
Supplement: Supplementary file 4 — Definitions of extent of resection and recurrence provided by authors. Supplementary material 4 (PDF 174 KB) [file 11060_2018_2901_MOESM4_ESM.pdf]

Online Resource 4: Definitions of extent of resection and recurrence/progression provided by study authors

	ALFORD ET AL [13]	DODGSHUN ET AL [14]	DORWARD ET AL [15]	KIM ET AL [16]	UDAKA ET AL [17]	VASSILYADI ET AL [18]	KORONES ET AL [12]
**GROSS TOTAL RESECTION**	No residual tumour	Absence of enhancing nodular elements determined on the immediate postoperative MR scan defined by radiologist	Lack of nodular enhancement on early postoperative MR imaging (i.e. within 48 hours of surgery)	Lack of nodular enhancement on imaging performed in the immediate postoperative period (within 3 days of the surgery) by a neuro-radiologist &2 neurosurgeons	NR	NR	NR
**SUB-TOTAL RESECTION**	Residual tumour	N/A	N/A	N/A	NR	NR	NR
**RECURRENCE**	NR	NR	Development of progressive, nodular enhancement on 2 successive follow-up images	Early nodular enhancement on imaging at 3-6 months	NR	NR	Ttumor which had completely responded to treatment and regrew
**PROGRESSION**	NR	N/A	N/A	N/A	25% increase in the maximal cross-sectional area documented on MRI	Residual lesion enlargement in all three dimensions compared to the previous MRI image	Residual tumor which progressed

Key: MRI: Magnetic resonance imaging; N/A: not applicable; NR: not reported

The utility of routine surveillance screening with magnetic resonance imaging (MRI) to detect tumour recurrence in children with low grade central nervous system (CNS) tumours: a systematic review

Journal of Neuro-oncology

Simon P. Stevens,[1] Caroline Main,[1] Simon Bailey,[2] Barry Pizer,[3] Martin English,[5] Robert Phillips,[6] Andrew Peet,[4] Shivaram Avula,[3] Sophie Wilne,[7] Keith Wheatley,[1] Pamela R. Kearns,[1,5] Jayne S.Wilson[1]

[1] Cancer Research UK Clinical Trials Unit (CRCTU), Institute of Cancer and Genomic Sciences, University of Birmingham, UK
[2] Sir James Spence Institute of Child Health, Royal Victoria Infirmary, Newcastle-Upon-Tyne
[3] Alder Hey Children's NHS Foundation Trust, Liverpool, UK
[4] Institute of Cancer and Genomic Sciences, University of Birmingham, UK
[5] Birmingham Women and Children's Hospital NHS Foundation Trust, Birmingham, UK
[6] Centre for Reviews and Dissemination (CRD), University of York, UK
[7] Queen's Medical Centre, Nottingham University Hospitals' NHS Trust, Nottingham, UK

Correspondence:
Jayne Wilson
UK; Tel: +441214149273
Email: j.s.wilson.1@bham.ac.uk
