# Supplementary material for: The utility of routine surveillance screening with magnetic resonance imaging (MRI) to detect tumour recurrence in children with low-grade central nervous system (CNS) tumours: a systematic review
Source: J Neurooncol. 2018 Jun 9;139(3):507–22. doi: 10.1007/s11060-018-2901-x (PMC6132973; doi:10.1007/s11060-018-2901-x)
Supplement: Supplementary file 5 — Quality appraisal of included primary studies. Supplementary material 5 (PDF 171 KB) [file 11060_2018_2901_MOESM5_ESM.pdf]

## Online Resource 5: Quality appraisal of included primary studies

| Study (year) [ref] | Study design | Selection bias | Detection bias | | | | |
|---|---|---|---|---|---|---|---|
| | | Is the study sample representative of the relevant population? | Are inclusion criteria explicit? | At study entry were participants at a similar timepoint in their disease progression? | Was follow-up long enough for important outcomes to occur? | Were outcomes assessed using objective criteria/blinding? | If sub-series comparisons are made is there sufficient information on sample distribution & prognostic factors? |
| **Low Grade Tumour studies** | | | | | | | |
| Alford et al (2016) [13] | Retrospective case series | + | + | + | + | + | NA |
| Dodgshun et al (2016) [14] | Retrospective case series | + | + | + | NR | + | NA |
| Dorward et al (2010) [15] | Retrospective case series | + | + | + | + | + | NA |
| Kim et al (2014) [16] | Retrospective case series | + | + | + | + | + | NA |
| Udaka et al (2013) [17] | Retrospective case series | + | + | + | + | + | + |
| Vassilyadi et al (2009) [18] | Retrospective case series | + | + | + | + | + | NA |
| **Mixed Grade Tumour studies** | | | | | | | |
| Korones et al (2001) [12] | Retrospective case series | + | + | + | NR | +/- | + |

**Key:** +: yes; -: no; +/-: Partial; NA: Not applicable; ?: Unclear

**The utility of routine surveillance screening with magnetic resonance imaging (MRI) to detect tumour recurrence in children with low grade central nervous system (CNS) tumours: a systematic review**

**Journal of Neuro-oncology**

Simon P. Stevens,[1] Caroline Main,[1] Simon Bailey,[2] Barry Pizer,[3] Martin English,[5] Robert Phillips,[6] Andrew Peet,[4] Shivaram Avula,[3] Sophie Wilne,[7] Keith Wheatley,[1] Pamela R. Kearns,[1,5] Jayne S.Wilson[1]

[1] Cancer Research UK Clinical Trials Unit (CRCTU), Institute of Cancer and Genomic Sciences, University of Birmingham, UK
[2] Sir James Spence Institute of Child Health, Royal Victoria Infirmary, Newcastle-Upon-Tyne
[3] Alder Hey Children's NHS Foundation Trust, Liverpool, UK
[4] Institute of Cancer and Genomic Sciences, University of Birmingham, UK
[5] Birmingham Women and Children's Hospital NHS Foundation Trust, Birmingham, UK
[6] Centre for Reviews and Dissemination (CRD), University of York, UK
[7] Queen's Medical Centre, Nottingham University Hospitals' NHS Trust, Nottingham, UK

**Correspondence:**
Jayne Wilson
UK; Tel: +441214149273
Email: j.s.wilson.1@bham.ac.uk
