# Supplementary material for: The utility of routine surveillance screening with magnetic resonance imaging (MRI) to detect tumour recurrence in children with low-grade central nervous system (CNS) tumours: a systematic review
Source: J Neurooncol. 2018 Jun 9;139(3):507–22. doi: 10.1007/s11060-018-2901-x (PMC6132973; doi:10.1007/s11060-018-2901-x)
Supplement: Supplementary file 6 — Study authors’ conclusions. Supplementary material 6 (PDF 224 KB) [file 11060_2018_2901_MOESM6_ESM.pdf]

Online Resource 6: Study authors' conclusions

Study	Authors' conclusions
**Alford et al (2016) [13]**	'…frequent MRI surveillance (every 3-6 months) may be employed in patients with CPAs until gross total resection is documented on two consecutive studies spaced ≥ 3 months apart. This will ensure adequate follow-up in the immediate postoperative period and when suspicious findings are present, but eliminate the unnecessary risks and costs of excessive long term imaging'.
**Dodgshun et al (2016) [14]**	'We report a large series of patients with PAs in whom GTR has been achieved. The cohort had a very low rate of progression and all progressions in the series were detected on surveillance MRI scans, with no clinical symptoms apparent and all were observed for at least one additional scan before receiving further treatment. This suggests that recurrences after GTR are rare and progress slowly.<br>We concur with the recommended schedule of surveillance MRI suggested by Kim et al (i.e. one contrast-enhanced MRI brain scan in the immediate postoperative period and at 3 months, followed by scans at 1, 2, 5, and 10 years from diagnosis) with the slight alteration of omitting the 10-year scan and replacing it with a scan between 3 and 4 years after resection. Adopting this schedule will result in significant time and cost savings for this group of patients at very low risk of tumor recurrence, while preserving their safety.'
**Dorward et al (2010) [15]**	'…we found that the presence of nodular tumor enhancement on the initial surveillance MR imaging study had predictive value for recurrent disease… However, our cases of delayed recurrence of GTR tumors in purportedly low-risks emphasize that the absence of abnormalities on surveillance imaging does not eliminate the risk of developing recurrent disease. Prospective studies will help refine risk stratification and will allow for the optimization of future postoperative surveillance protocols'.
**Kim et al (2014) [16]**	'…given the small but real risk of recurrence even after GTR, we recommend a schedule of long-term postoperative surveillance MRI. Our recommended protocol has clear advantages over the current regimen at Boston Children's Hospital due to our findings that most recurrences in this group are clinically asymptomatic and generally indolent, allowing us to substantially decrease the number of MR scans necessary to safely follow up these patients. We recommend the following postoperative MRI schedule: (postoperative time = time 0) 1-3 days, 3 months, 1 year, 2 years, 5 years, and 10 years. This reduces the number of MR scans in the first 5 years following GTR from 10 to 5…. The proposed schedule carries a clear financial benefit in terms of health care costs, and may also lessen the psychological burden of frequent scans in this pediatric population.'
**Korones et al (2014) [12]**	'…the proportion of children in our study who were asymptomatic at the time of MRI-documented recurrence is high. However, an asymptomatic recurrence was detected in only one of 24 surveillance scans, and the survival of asymptomatic children was not improved as a result. Our low rate of detection of recurrences and the lack of improvement in outcome in spite of asymptomatic detection suggest that we can decrease the frequency of surveillance.'
**Udaka et al (2013) [17]**	'Our findings suggest that long-term monitoring with limited MRI sequences may be warranted, even in asymptomatic children who have undergone GTR, as many as 5 years after LGG diagnostic/treatment. The sole use of T1-weighted MRI before and after Gd administration may be sufficient to diagnosis LGG progression or recurrence, and the matter is worthy of further validation in a larger multi--institutional cohort.'<br>'The authors' findings support the need for routine neuroimaging in a subset of children with LGGs, even when gross total resection has been achieved, up to 5 years post-diagnosis.'
**Vassilyadi et al (2009) [18]**	'In conclusion, the results of this study support that pediatric patients with either pilocytic or non-pilocytic cerebellar astrocytomas who have undergone gross and radiologic total resection may not benefit from routine surveillance neuroimaging, primarily because of low recurrence likelihood. Those patients in whom STR is obtained are expected to benefit from surveillance of the residual disease….with further work aimed at exploring the schedule of such follow-up. The benefits of developing a more stringent surveillance program following complete resection of low-grade astrocytic tumors include more cost-effective health care delivery and lesser exposure of pediatric patients to general anaesthesia'.

The utility of routine surveillance screening with magnetic resonance imaging (MRI) to detect tumour recurrence in children with low grade central nervous system (CNS) tumours: a systematic review

Journal of Neuro-oncology

Simon P. Stevens,[1] Caroline Main,[1] Simon Bailey,[2] Barry Pizer,[3] Martin English,[5] Robert Phillips,[6] Andrew Peet,[4] Shivaram Avula,[3] Sophie Wilne,[7] Keith Wheatley,[1] Pamela R. Kearns,[1,5] Jayne S.Wilson[1]

[1] Cancer Research UK Clinical Trials Unit (CRCTU), Institute of Cancer and Genomic Sciences, University of Birmingham, UK
[2] Sir James Spence Institute of Child Health, Royal Victoria Infirmary, Newcastle-Upon-Tyne
[3] Alder Hey Children's NHS Foundation Trust, Liverpool, UK
[4] Institute of Cancer and Genomic Sciences, University of Birmingham, UK
[5] Birmingham Women and Children's Hospital NHS Foundation Trust, Birmingham, UK
[6] Centre for Reviews and Dissemination (CRD), University of York, UK
[7] Queen's Medical Centre, Nottingham University Hospitals' NHS Trust, Nottingham, UK

Correspondence:
Jayne Wilson
UK; Tel: +441214149273
Email: j.s.wilson.1@bham.ac.uk
